# Development and psychometric properties of a questionnaire to evaluate sustainable waste separation behavior and environmental health promotion

**DOI:** 10.1186/s42506-021-00090-1

**Published:** 2021-10-26

**Authors:** Mahnaz Solhi, Esmat Heydari, Leila Janani, Mahdi Farzadkia

**Affiliations:** 1 Department of Health Education and Health Promotion, School of Public Health, Iran University of Medical Sciences, Tehran, Iran; 2 Department of Biostatistics, School of Public Health, Iran University of Medical Sciences, Tehran, Iran; 3 Department of Environmental Health Engineering, School of Public Health, Iran University of Medical Sciences, Tehran, Iran

**Keywords:** Waste separation, Community-based social marketing, Questionnaire, Factor analysis

## Abstract

**Background:**

Assessment of benefits and barriers of waste separation at source is necessary to identify the effective factors in this field. This study aims at designing and validation of a questionnaire assessing the barriers and benefits of waste separation at source from the viewpoint of women based on the community-based social marketing model in Genaveh Township, Bushehr, Iran

**Methods:**

In phase 1, a literature review and focus group discussion were conducted to identify the barriers and benefits of waste separation at the source and to design the items and questionnaires. In phase 2, a psychometric evaluation was performed, including face validity, content validity, structural validity, and reliability. Data were analyzed by SPSS and R software.

**Results:**

Out of 56 initial items, seven items with the content validity ratio less than 0.56 were removed, and one with the content validity index less than 0.79 was reviewed. Content validity ratio of the questionnaire was 0.782 and content validity index 0.957. The results of exploratory factor analysis showed that the five and seven-factor models showed good fit. Considering the possible existence of some items in several factors, confirmatory factor analysis was used in the next step. Finally, two items were removed and two others were displaced. The reliability of the instrument was confirmed by internal consistency (*α* = 0.92) and its stability by the test-retest (ICC = 0.83).

**Conclusions:**

The valid and reliable 48-item questionnaire is a suitable instrument for assessing the barriers and benefits of waste separation at source from the viewpoint of women based on the community-based social marketing model in Genaveh Township, Bushehr.

## Introduction

The global crisis of increasing waste production is emerging due to the rapid growth of population and has affected most cities around the world [1]. Increasing waste generation and its improper disposal can lead to crises such as water and soil pollution, greenhouse gas emissions, and negative impacts on the quality of life [2–5].

Currently, in many countries, new methods of waste separation and recycling are used to organize and manage municipal waste and thereby solve environmental problems [6]. In waste management, two methods are preferable to others, i.e., waste reduction and separation at source [7]. Waste separation at source is the process of separating dry wastes from wet wastes. This method has been exploited with the aim of recycling a significant portion of the wastes to be reused in the production, resulting in a significant reduction in the cost of waste collection and disposal compared to the conventional methods. In addition, the advantages of waste separation at source over other methods include economic benefits, saving the land needed for waste disposal and reducing the cost of maintenance of compost plants and high quality compost production [8, 9].

In Iran, only 8% of wastes are recycled and the rest is buried in an unhygienic manner [10]. The main reason for the failure of recycling programs is the lack of citizen participation, failure to properly and regularly perform waste separation at source by the contractors, and organizational problems at the management level. Meanwhile, the lack of citizen participation and improper planning of waste management programs are the most important reasons for failure of the waste separation [11, 12].

In the program of waste separation at source, that requires the maximum participation of the citizens, modern and advanced technology does not play a significant role. Success in this management methodology depends on sound policy making, making proper educational and cultural programs for the general population, development of specific laws and regulations, and establishment of the necessary infrastructure by the authorities [2, 12]. Identifying the reasons for not cooperating and contributing to the program of waste separation at source by the citizens is useful in providing effective and practical solutions to improve the waste management system in cities, especially for separating recyclable wastes at source.

A theory-based study is needed to understand and identify the mechanisms responsible for recycling behaviors [5]. In this study, a community-based social marketing model (CBSM) was used to design and validate the questionnaire of barriers and benefits of waste separation by women. CBSM is a social marketing technique that aims to influence the behavior by a direct communication at the person-to-person or community level. This eliminates barriers and at the same time increases the perceived benefits of behavior. The CBSM process consists of 5 steps: (1) behavior selection, (2) identifying barriers and benefits, (3) developing strategies, (4) piloting the program (implementing interventions), and (5) implementing the program. CBSM uses 7 tools to perform interventions [13]. Because the purpose of this study was to design instrument based on CBSM, only steps 1 and 2 were implemented in this study.

There are two main reasons for using CBSM in this study: (1) Topics such as waste reduction and waste separation, increasing energy efficiency, reducing water consumption, and changing transportation patterns are generally sustainable projects that require sustainable behaviors. Expedient models and theories should be used to design and implement sustainable projects. Today, CBSM is used to design and implement sustainable projects [14–18]. (2) Compared to using the existing instruments, the constructions of instruments will be the most effective approach when the concept being analyzed is affected by the contexts of the community under study [19], something that has been well considered in the CBSM.

According to steps 1 and 2 in the community-based social marketing model, after selecting the behavior, one should identify the barriers and benefits of the relevant behavior first through literature reviews and then by deep and qualitative studies (focus group discussion or interviewing) in the study population. After qualitative study, concepts and items are extracted and used to design study instruments [13].

Considering the importance of recognizing the barriers and benefits of waste separation at source, any studies in this area requires a dedicated standard instrument. The literature review shows that although there have been instruments to assess knowledge, attitudes, or factors affecting waste separation, no instruments were found to measure its barriers and benefits. On the other hand, since waste separation is a community-based variable and is influenced by cultural and social factors of the society, its measurement requires access to an instrument with desirable psychometric properties and commensurate with social, cultural, and structural context of the community. The present study aimed to design and validate the questionnaire of benefits and barriers of waste separation at source from the viewpoint of women based on the CBSM in Genaveh.

## Methods

The present study is part of a research project on design, implementation, and evaluation of an intervention based on CBSM on waste separation at source from the viewpoint of women in Genaveh, Bushehr. It is a Psychometric Research performed in Genaveh in 2019, which obtained the ethical code (IR.IUMS.REC.1397.642) from the Ethics Committee of the Faculty of Health of Iran University of Medical Sciences. Based on the CBSM, for sustainable behavior to be widely accepted, the barriers and benefits of a behavior need to be identified through a combination of community-based research methods [13]. Therefore, in order to prepare the questionnaire entitled “Investigating the barriers and benefits of waste separation at source based on the community-based social marketing model,” we first designed the questionnaire items through literature review and focus group discussion and then made a psychometric analysis on the questionnaire. The details and steps of the procedure are described in two separate phases.

### Phase 1: Item generation and questionnaire development

In order to design the items, first, a literature review and then a qualitative study were done.

#### Literature review

A literature review was conducted to identify the barriers and benefits of waste separation at source within the country since 2001 using the Persian keywords equivalent to “waste separation, recycling, and CBSM” in the SID, Iranmedex, Irandoc databases. Also, studies conducted overseas were reviewed since 1990 using these English keywords: Benefits and Barriers to Recycling, Waste Diversion, Recycling, Community Based Social Marketing, Waste Reduction, Recycling Behavior, and Determinants of Recycling in PubMed Databases, Science direct, Google Scholar, Ovid, and Scopus Search. The studies were divided into two parts: studies on waste separation barriers and studies on waste separation benefits. The concepts and items related to the barriers and benefits of waste separation at source were extracted from the reviewed studies and used for the next step.

#### Qualitative study

Also, because waste separation in a community depends on the various cultural, social, economic, and infrastructure factors of that community, we performed a focus group discussion to identify the barriers and local benefits of waste separation at source. The subjects were divided into two groups: those who performed waste separation and those who did not. Some of them were randomly contacted and invited to participate in the discussion. Three sessions of focus group discussion were held for each group (6 sessions in total for both groups) with 8 individuals attending each session, until the data saturation was obtained, so that no new items were presented by women. Items of the questionnaire were obtained by the literature review and the highest frequencies referred to by the participants in the group discussion. Written consent was obtained from participants in group discussions and ethical considerations were taken into account.

### Phase 2: Psychometric properties of the instrument

In this phase of the study, the face validity, content validity, and construct validity of the instrument were assessed. Reliability of the questionnaire was assessed in terms of internal consistency and stability.

#### Qualitative content validity

To evaluate the validity of qualitative content (review of content and structure of items), the experts’ panel was used. The selection of the experts’ panel was done based on the topic and field of the research with the opinion of the professors and consultants present in the research team. Hence, based on the topic—Evaluating the Barriers and Benefits of Waste Segregation at Source Based on the Community-Based Social Marketing Model—which is an interdisciplinary topic, the views of environmental health experts (3 experts), health education (6 experts), and epidemiology (3 experts) were used so that the views of experts in various fields related to the subject may contribute to the evaluation of the questionnaire. In order to perform the qualitative validity of the content, first, the items, research objectives, target groups, and the subject of the research were fully provided to the experts so that after carefully studying the items, they suggest their corrective views in detail and in writing regarding grammar observance, use of appropriate words, placement of items in their proper place, and appropriate scoring [20]. Based on the views of experts, the necessary changes were made in the questionnaire items.

#### Quantitative content validity

Quantitative content validity was analyzed using content validity ratio (CVR) and content validity index (CVI). In this regard, 12 participants were selected for the panel of experts to judge the quantitative content validity of the items. These included faculty members in environmental health (*n* = 3) and health education and health promotion (*n* = 6) and epidemiology (*n* = 3). To determine the content validity ratio, the panel of experts was asked to examine each item on a three-point scale (necessary, useful but not necessary, not necessary). In the CVR, the responses were calculated based on the following formula [21], in which the number of experts who selected the item of essential is indicated by (*n*_*E*_) and the total number of evaluators by (*N*).

$$ CVR=\frac{n_{E-\frac{N}{2}}}{\frac{N}{2}} $$

For this purpose and based on the Lawshe chart [22], questions whose content validity ratios were equal to or greater than 0.56 (based on evaluations of a 12-expert panel) were considered significant (*p* < 0.05) and remained in the questionnaire.

Then, in order to evaluate the CVI, a panel of experts was asked to evaluate the questionnaire by three indices (simplicity, clarity, and relevance) in a 4 point Likert scale (from 1 minimum to 4 maximum points). The CVI index was calculated using the following formula [23], taking into account the total agree scores for each item that ranks 3rd and 4th (highest score).

$$ CVI=\frac{The\ ratio\ of\ the\ number\ of\ evaluators\  who\  gave\ the\ score\ of\ 3\  and\ 4\  to\ the\ item}{Total\ number\ of\ evaluators\ } $$

Then, considering that the number of experts was 12 and based on the Lawshe chart [22], items with a score above 0.79 were considered significant. A score between 0.7 and 0.79 is questionable and needs to be corrected and revised. Less than 0.7 is unacceptable and will be removed [23].

#### Qualitative face validity

To assess the qualitative face validity of the questions, the questionnaire was randomly distributed among 15 women living in Genaveh and their opinions about difficulty level (difficulty understanding words and phrases), relevancy (compatibility and relevancy of phrases with the dimensions of the questionnaire), and ambiguity (probability of misinterpretation of expressions or inaccuracies in verbal meanings) were inquired to correct the questionnaire items from this perspective and remove or correct the inappropriate items [21].

#### Quantitative face validity

After correcting the items based on the comments of the target group, the impact score was used to reduce and eliminate inappropriate items and determine the importance of each item. To determine quantitative face validity, the Impact Score for each item was calculated using the following formula: Impact Score = Frequency (%) × Importance. In evaluating the method of impact score, by frequency in terms of percentage, we mean the number of people who gave a score of 4 and 5 to the item, and by importance, we mean the mean score of importance based on the Likert scale. The item with a score equal to or greater than 1.5 is retained and other items are omitted [24, 25]. Accordingly, the items were compiled as very important (score 5), important (4), averagely important (3), slightly important (2), and not important at all (1). The quantitative face validity was calculated for each item using the item impact formula after scoring by 15 women in the target group [24].

#### Construct validity

In exploratory and confirmatory factor analysis, the minimum sample size should be 200 people and in addition, 5 to 10 samples can be selected for each item [25, 26]. Accordingly, in this study, for construct validity, the sample size per item (50 items) was considered to be 6 people where a total of 300 people were selected by available sampling. The questionnaire consisted of 50 items in a five-point Likert scale ranging from “strongly agree” to “strongly disagree.” Descriptions of the purpose and how to complete the questionnaire were provided at the beginning of the questionnaire. Inclusion criteria for this phase of the study consisted of women living in Genaveh, having minimum literacy (ability to read and write), age over 18. Data were collected by one of the researchers (environmental health expert) who was familiar with the research method and sampling principles. For this purpose, the researcher went to health centers and clinics in Genaveh. He invited the women who had the inclusion criteria and who were sitting in the waiting room of the clinic to take part in the study after he described the objectives of the study and took their informed consent. The questionnaire was provided to the women to be completed in a self-report manner. The data was collected and analyzed using the SPSS software (version 22, SPSS Inc., Chicago, IL, USA).

To evaluate the construct validity of this study, an exploratory factor analysis [26] was performed to identify patterns in items. The five- and seven-factor models showed good fit. Given the possibility of the presence of some items in several factors, the confirmatory factor analysis was used in the next phase based on the model that served as the basis for item extraction.

A single confirmatory factor analysis was first performed for each construct, and items that did not fit the construct were identified. Confirmatory factor analysis model was implemented using the R software based on diagonal weighted least squares robust estimation (DWLS) fitting method and robust estimation method of weighted least squares means and variance adjusted (WLSMV) type which are suitable for ordinal rather than continuous data [27]. Finally, the structure of the questionnaire was approved.

#### Reliability

Test-retest method was used to assess the reliability of the items in the questionnaire. For this purpose, the questionnaire was given to 30 women twice within a 10-day interval. The data was collected and analyzed using the SPSS software (version 22, SPSS Inc., Chicago, IL, USA), and the intra class correlation coefficient was calculated. Also, to investigate the internal consistency of the items, the questionnaire was completed by 300 women in the target group and after entering the data into the SPSS software; the Cronbach’s alpha was calculated. The Cronbach’s alpha must be between 0.70 and 0.80 to make a good internal consistency [28].

## Results

### Validity

Evaluating the qualitative content validity of the vocabulary used, grammar, proper placement, and the structure and concept of each item, the panel of experts commented on these areas. Accordingly, the necessary changes were made to the questionnaire items. As a result, several items were revised and necessary changes were made. The results of content validity ratio showed that 50 items scored above 0.56 and were retained in the questionnaire, and 6 items (8, 25, 36, 43, 44, and 45) scored less than 0.56 and were eliminated from the questionnaire. Also, the results of content validity index showed that all items scored above 0.79, except for item 20, which achieved 0.75 in terms of clarity, and was therefore revised (Table 1).

**Table 1:** Results of face and content validity of the instrument

No.	Item	Impact score	CVI			CVR
			Simplicity	Relevancy	Clarity	
1	Waste separation requires lots of space.	3.04	0.91	1	1	1
2	Because recycling is poor, waste separation at home is not important and effective.	1.62	1	1	1	0.66
3	Because waste separation is of little economic value to the family, I do not separate wastes.	1.56	1	1	0.91	1
4	Because collection of wastes at home is time consuming, I do not separate wastes.	1.56	1	1	0.91	1
5	Because I need a separate trash can in the kitchen for separation, I do not separate wastes.	1.56	1	1	1	0.83
6	Moving the recyclables outdoors is difficult, especially in buildings without elevator.	1.56	1	1	1	0.66
7	Collecting recyclables causes an unpleasant odor.	1.62	1	1	0.91	0.66
8	Waste separation is difficult for apartment residents due to the small space and the shared staircase, elevator and yard.	1.68	0.91	0.91	0.83	0.5
9	Collecting recyclables causes disorder indoors and outdoors.	1.92	1	1	0.83	0.83
10	I like to separate wastes but I don't have the required space.	2.8	1	1	0.91	0.83
11	I get confused to figure out which trash can each type of waste should be put into.	1.51	1	1	0.83	0.83
12	Women do not have enough time to separate wastes.	1.74	1	1	0.91	0.83
13	Due to non-cooperation of the municipality in the collecting recyclables, I do not separate the wastes.	1.8	1	1	0.91	1
14	Due to the lack of a comprehensive recycling plan, even if I separate the wastes, the wastes are still disposed of together and the separation would be useless.	1.8	0.83	1	0.83	0.83
15	Because the refuse collectors are not justified in terms of recycling plan, even if I separate the wastes, the wastes are still disposed of together and the separation would be useless.	2.24	0.83	1	0.83	0.83
16	Because of the lack of recycling bins in neighborhoods or apartment complexes, I do not separate wastes.	2.4	1	1	0.91	1
17	Because refuse collectors do not come at my door to collect recyclables, I do not separate wastes.	1.86	1	1	0.83	0.66
18	I do not know the methods and phases of waste separation.	1.83	1	1	0.91	0.66
19	I do not know which part of the dry waste is recyclable and needs to be separated.	1.5	1	1	0.91	0.83
20	Waste separation at home is unpleasant to me.	1.51	0.91	0.91	0.75	0.66
21	I am not in the mood for waste separation.	1.62	1	1	0.91	0.83
22	I think separating waste at home is unnecessary and pointless.	1.56	1	1	0.91	1
23	Daily separation of waste is boring for me.	1.8	1	1	0.83	0.83
24	Busyness makes it impossible for me to separate wastes.	1.51	1	1	0.91	1
25	After a long working day and due to numerous house chores, waste separation is a difficult, daunting task.	1.62	0.91	0.91	0.91	0.33
26	I do not separate wastes because it is beyond my dignity.	1.62	0.91	1	0.91	0.66
27	Although I would like to separate wastes at home, I do not have enough time to do it.	2.38	0.91	1	0.91	0.66
28	Although I have enough time, I consider waste separation a waste of time and energy.	1.62	0.91	1	0.91	0.66
29	Environmental protection is not important to me.	3.87	0.91	1	0.91	0.83
30	I do not care what will happen to the collected wastes.	1.5	0.91	1	0.91	0.66
31	In my opinion, waste separation is the responsibility of certain organizations, not the citizens, and should be performed after the collection.	1.68	0.83	0.83	0.83	0.66
32	It is easier to carry unseparated wastes outdoors, so I do not separate wastes.	2.88	0.91	1	1	1
33	Because more garbage bags are required to be used at home, I do not separate wastes.	2.17	1	1	1	1
34	Because of the high cost of the garbage bag, I do not separate wastes.	1.83	1	0.91	1	0.66
35	I do not separate wastes because of laziness.	1.68	1	1	1	0.66
36	I do not separate wastes because it is troublesome.	1.62	1	1	1	0.33
37	Because I do not receive any money or goods in turn for the delivery of recyclables, I do not separate wastes at home.	2.38	1	1	0.91	0.83
38	My spouse or family members do not participate in waste separation, so I do not separate wastes.	1.62	1	1	0.91	1
39	Waste separation only by me does not have much impact on improving waste management.	1.56	1	1	0.83	1
40	The economic problems in our country take away the opportunity to think about the environment and its problems.	1.83	1	1	0.91	0.66
41	The real reason that authorities encourage people to separate wastes at home is to make more money for themselves.	1.68	1	1	0.91	1
42	Waste separation at home can reduce the pollution of the urban environment.	1.74	1	1	0.91	1
43	I separate wastes at home because I am worried about cutting down trees and destroying forests.	2.38	1	0.91	0.83	0.33
44	I separate wastes at home because I am worried about groundwater contamination.	2.96	1	0.91	0.91	0.33
45	I separate wastes at home because I'm worried about air pollution.	3.04	1	0.91	0.91	0.33
46	By separating wastes I want to retain a better world for my children and future generations.	3.96	1	1	0.91	1
47	To protect the environment, I always spend time separating wastes.	3.04	1	1	1	1
48	I get paid for the recyclables I deliver, so I always separate wastes at source.	1.74	1	1	1	0.83
49	If I do not separate wastes, I will seem to be irresponsible.	3.04	1	1	1	0.83
50	I performed waste separation so that they are reused as raw materials in the production cycle of factories.	2.24	1	1	1	0.83
51	I want to promote waste separation culture among others (children, other family members, neighbors, relatives, etc.) by separating waste at home.	1.68	1	1	0.91	0.83
52	I want to help create jobs in the community by separating waste at home.	1.83	0.91	0.91	1	0.83
53	I want to help reduce the amount of wastes in the city by separating waste at home.	2.31	1	1	1	1
54	I want to help postpone the filling of dumping areas by separating waste at home.	1.56	0.91	0.91	0.83	0.66
55	I help reduce the cost of waste transportation by the municipality through separating waste at home.	1.5	1	1	1	0.83
56	I intend to be more serious about waste separation at home in the future.	1.56	1	1	1	0.83

### Face validity

The results of qualitative validity assessment by the respondents indicated the relevance of the items to the subject under study, correct understanding and no ambiguity and difficulty in understanding the items. Quantitative face validity also showed that the item impact score was 1.5 to 3.96 (Table 1).

### Construct validity

To assess construct validity, a questionnaire consisting of 50 items was completed by 300 women on a 5-point Likert scale ranging from strongly agree to strongly disagree. The mean age of participants was 30.16 ± 8.07 years. In total, 76.3% of the women were housewives and 77.3% had a high school diploma. Table 2 shows the demographic data of the respondents for the factor analysis step (Table 2).

**Table 2:** Demographic characteristics of participant in exploratory/confirmatory factor analysis (*n* = 300)

Variables	Groups	*n*	%	Mean ± SD
Age	< 30 years old	176	58.7	30.16 ± 8.07
	30-40	93	31	
	41-60	31	10.3	
Marital status	Single	69	23	-
	Married	228	76	
	Divorced/widow	3	1	
Job	House keeper	202	67.3	-
	Clerk	29	9.7	
	Worker	1	.3	
	student	34	11.3	
	Other job	34	11.3	
Education level	≤ 12th (grade)	68	22.7	-
	> 12th (grade)	232	77.3	
Type of home	House	240	80	-
	The apartment	60	20	
Number of family members	-	-	-	4.06 ± 1.40

Construct validity results showed that after exploratory factor analysis, 5- and 7-factor models were recognized as having a good fit. Given the possibility of the presence of some items in several factors, the confirmatory factor analysis was used in the next phase based on the model that served as the basis for item extraction. Taking into account the structure proposed by McKenzie-Mohr to categorize the barriers and benefits of sustainable community-based social marketing behavior, the questionnaire items were categorized into five-barrier factors and one-benefit factor of waste separation, as shown in Table 3.

**Table 3:** Confirmatory factor analysis results

Factors	Items
Structural barriers	Waste separation requires lots of space. Because I need a separate trash can in the kitchen for separation, I do not separate wastes. Moving the recyclables outdoors is difficult, especially in buildings without elevator. Collecting recyclables causes an unpleasant odor. Collecting recyclables causes disorder indoors and outdoors. I like to separate wastes but I do not have the required space. Due to non-cooperation of the municipality in the collecting recyclables, I do not separate the wastes. Due to the lack of a comprehensive recycling plan, even if I separate the wastes, the wastes are still disposed of together and the separation would be useless. Because the refuse collectors are not justified in terms of recycling plan, even if I separate the wastes, the wastes are still disposed of together and the separation would be useless. Because of the lack of recycling bins in neighborhoods or apartment complexes, I do not separate wastes. Because refuse collectors do not come at my door to collect recyclables, I do not separate wastes. It is easier to carry unseparated wastes outdoors, so I do not separate wastes. Because more garbage bags are required to be used at home, I do not separate wastes. Because of the high cost of the garbage bag, I do not separate wastes.
Inadequate knowledge,	I do not know the methods and phases of waste separation. I do not know which part of the dry waste is recyclable and needs to be separated.
Forgetting the act	I get confused to figure out which trash can each type of waste should be put into.
Benefit	Waste separation at home can reduce the pollution of the urban environment. By separating wastes, I want to retain a better world for my children and future generations. I performed waste separation so that they are reused as raw materials in the production cycle of factories. I want to help create jobs in the community by separating waste at home. I want to help reduce the amount of wastes in the city by separating waste at home. I want to help postpone the filling of dumping areas by separating waste at home. I help reduce the cost of waste transportation by the municipality through separating waste at home.
Inadequate social pressure	My spouse or family members do not participate in waste separation, so I do not separate wastes. Waste separation only by me does not have much impact on improving waste management. If I do not separate wastes, I will seem to be irresponsible. I want to promote waste separation culture among others (children, other family members, neighbors, relatives, etc.) by separating waste at home.
Inadequate motivation	Because recycling is poor, waste separation at home is not important and effective. Because waste separation is of little economic value to the family, I do not separate wastes. Because collection of wastes at home is time consuming, I do not separate wastes. Women do not have enough time to separate wastes. Waste separation at home is unpleasant to me. I am not in the mood for waste separation. I think separating waste at home is unnecessary and pointless. Daily separation of waste is boring for me. Busyness makes it impossible for me to separate wastes. I do not separate wastes because it is beyond my dignity. Although I would like to separate wastes at home, I do not have enough time to do it. Although I have enough time, I consider waste separation a waste of time and energy. Environmental protection is not important to me. I do not care what will happen to the collected wastes. In my opinion, waste separation is the responsibility of certain organizations, not the citizens, and should be performed after the collection. I do not separate wastes because of laziness. Because I do not receive any money or goods in turn for the delivery of recyclables, I do not separate wastes at home. The economic problems in our country take away the opportunity to think about the environment and its problems. The real reason that authorities encourage people to separate wastes at home is to make more money for themselves. I get paid for the recyclables I deliver, so I always separate wastes at source. I intend to be more serious about waste separation at home in the future.

A single confirmatory factor analysis was first performed for each construct, and items that did not fit the construct were identified. At this stage, items 11, 49, 51, and 56 were incompatible with the constructs presented. After consulting with the panel of experts, items 49 and 56 were omitted, item 51 was placed in group 4, and item 11 in group 6 (Table 4). All loadings were significant (Table 5).

**Table 4:** Final questionnaire

Factors	Items
Structural barriers	Waste separation requires lots of space. Because I need a separate trash can in the kitchen for separation, I do not separate wastes. Moving the recyclables outdoors is difficult, especially in buildings without elevator. Collecting recyclables causes an unpleasant odor. Collecting recyclables causes disorder indoors and outdoors. I like to separate wastes but I do not have the required space. Due to non-cooperation of the municipality in collecting recyclables, I do not separate the wastes. Due to the lack of a comprehensive recycling plan, even if I separate the wastes, the wastes are still disposed of together and the separation would be useless. Because the refuse collectors are not justified in terms of recycling plan, even if I separate the wastes, the wastes are still disposed of together and the separation would be useless. Because of the lack of recycling bins in neighborhoods or apartment complexes, I do not separate wastes. Because refuse collectors do not come at my door to collect recyclables, I do not separate wastes. It is easier to carry unseparated wastes outdoors, so I do not separate wastes. Because more garbage bags are required to be used at home, I do not separate wastes. Because of the high cost of the garbage bag, I do not separate wastes.
Inadequate knowledge	I do not know the methods and phases of waste separation. I do not know which part of the dry waste is recyclable and needs to be separated.
Benefit	Waste separation at home can reduce the pollution of the urban environment. By separating wastes, I want to retain a better world for my children and future generations. I performed waste separation so that they are reused as raw materials in the production cycle of factories. I want to help create jobs in the community by separating waste at home. I want to help reduce the amount of wastes in the city by separating waste at home. I want to help postpone the filling of dumping areas by separating waste at home. I help reduce the cost of waste transportation by the municipality through separating waste at home. I want to promote waste separation culture among others (children, other family members, neighbors, relatives, etc.) by separating waste at home.
Inadequate social pressure	My spouse or family members do not participate in waste separation, so I do not separate wastes. Waste separation only by me does not have much impact on improving waste management.
Inadequate motivation	Because recycling is poor, waste separation at home is not important and effective. Because waste separation is of little economic value to the family, I do not separate wastes. Because collection of wastes at home is time consuming, I do not separate wastes. Women do not have enough time to separate wastes. Waste separation at home is unpleasant to me. I am not in the mood for waste separation. I think separating waste at home is unnecessary and pointless. Daily separation of waste is boring for me. Busyness makes it impossible for me to separate wastes. I do not separate wastes because it is beyond my dignity. Although I would like to separate wastes at home, I do not have enough time to do it. Although I have enough time, I consider waste separation a waste of time and energy. Environmental protection is not important to me. I do not care what will happen to the collected wastes. In my opinion, waste separation is the responsibility of certain organizations, not the citizens, and should be performed after the collection. I do not separate wastes because of laziness. Because I do not receive any money or goods in turn for the delivery of recyclables, I do not separate wastes at home. The economic problems in our country take away the opportunity to think about the environment and its problems. The real reason that authorities encourage people to separate wastes at home is to make more money for themselves. I get paid for the recyclables I deliver, so I always separate wastes at source. I get confused to figure out which trash can each type of waste should be put into.

**Table 5:** Model fitting statistics using the R software to validate structural constructs

Model fitting indicators	RMSEA	Percent confidence interval	*P* value RMSEA	SRMR	CFI	TLI	Model fit test statistic	Degrees of freedom	Chi-square/df
Values	0.052	0.048, 0.055	0.214	0.088	0.930	0.926	2387.677	1070	2.23

*RMSEA* root mean square error of approximation, *SRMR* residual square mean root (standardized), *CFI* comparative fit index, *TLI* Tucker-Lewis index, *df* degrees of freedom, *χ2* chi-square, *χ2/df* chi-square/df

### Reliability

Results of questionnaire reliability analysis showed that intra class correlation coefficient was 0.83. The internal consistency of the items was obtained using Cronbach’s alpha coefficient as 0.92.

## Discussion

McKenzie-Mohr classifies identified barriers to sustainable behavior in five factors, including inadequate motivation, forgetting the act, inadequate social pressure, inadequate knowledge, and structural barriers [13]. Since the present study was designed by the CBSM, the barriers and benefits associated with waste separation at source were initially categorized into five factors of barriers and one factor of benefit. Because the number of items of the factor of “forgetting the act” did not meet the requirements to become a separate factor, this item was merged with an “inadequate motivation” factor and the final questionnaire was designed with four factors of barriers and one factor of benefits of waste separation from the viewpoint of women. Meanwhile, Babazadeh et al. categorized the barriers of waste separation into four factors of problems of the waste collection system, lack of citizen responsibility, inadequate knowledge, and expectation of incentives [11]. In another study, the barriers to waste separation were classified into four categories, including situational, behavioral, knowledge, and attitude barriers [29]. In other research also categorized the barriers to waste separation at source into six factors, including inadequate facilities, inadequate attitude, inadequate knowledge, inadequate commitment, uncomfortableness, and lack of enforcement [30]. The reason for this difference in classifications is the difference in the methodology of the studies.

A review of the studies on validation of the questionnaire of waste separation at source based on the behavioral theories revealed that so far only the planned behavior theory (TPB) has been used in this regard, as Erami et al. made a study on strategies to increase the participation of housewives in solid waste separation at source and devised a questionnaire consisting of 8 constructs based on the TPB [5]. Also, in another study entitled “Use of the Planned Behavior Theory to Determine Recycling Behaviors in Bristol City, England,” a questionnaire consisting of six constructs was designed and validated based on TPB [31]. However, no CBSM-based questionnaire has been designed so far, and this is the distinguishing factor of the present study.

The results of the validation of the instrument designed in this study showed that the questionnaire assessing the barriers and benefits of waste separation at the source from the viewpoint of women based on CBSM were valid and reliable, and the Cronbach’s alpha for the whole scale was 0.92. Erami et al. conducted a study on strategies to increase the participation of housewives in solid waste separation at source and reported a Cronbach’s alpha of 0.87 [5]. Several other studies reported lower levels of Cronbach’s alpha [31–35]. These results show that the designed instrument has higher internal consistency than the mentioned study instrument. This can be due to the use of a combination of methods, such as literature reviews and qualitative studies to extract study items of the instrument.

Test-retest results also showed that the items in the questionnaire had a good correlation (ICC = 0.83). This suggests that using a questionnaire in different situations can yield relatively similar results. Erami et al. reported similar results (ICC = 0.89) [5].

Assessment of content validity ratio using panel of experts showed that 50 items scored above 0.56 and were retained in the questionnaire and 6 items scored less than 0.56 and were eliminated from the questionnaire. Also, the results of content validity index evaluation showed that all items scored above 0.79, except for the item 20 which achieved 0.75 in terms of clarity and was revised.

The results of exploratory factor analysis showed that the 5- and 7-factor models were of good fit. Accordingly, given the possibility of the presence of some items in several factors, the confirmatory factor analysis was used in the next phase. At this stage, items 11, 49, 51, and 56 were incompatible with the constructs presented, after consulting with the panel of experts, items 49 and 56 were omitted, item 51 was placed in group “benefits of waste separation,” and item 11 in group “inadequate motivation.” As a result, the number of items in the final questionnaire was reduced to 48 items.

To discuss the relevancy of the confirmatory factor analysis, we need to consider the indices of this model. It has been suggested that the RMSEA values less than 0.08 are acceptable. The RMSEA value in this study is 0.052 indicating an acceptable value. In the case of the CFI index, values greater than 0.9 are acceptable. This value is 0.93 in the present study, which is acceptable [36]. Also, the Chi-square/df index below 3 is acceptable, and it is 2.23 in the present study [37].

### Limitations of the study

The limitations of this study, data were obtained based on a self-report questionnaire, which leads to probable bias in the results. Also, data of this study was collected from a sample of women in Genaveh Township. Therefore, the results of this study cannot be generalized to all groups of women in Iran. Performing similar study in other groups of women in other geographical areas of Iran is suggested.

## Conclusion

In this study, a scientific instrument was developed comprised 48 items to measure barriers and benefits of waste separation at source from the viewpoint of women, which can be used by the municipal service managers and researchers in this area to investigate barriers and benefits of waste separation from the viewpoint of women. Although the results of this study confirm the psychometric properties of the designed instrument, similar studies in other communities are needed to generalize the findings of this study.

## Data Availability

Data will be available on reasonable request. Please contact the corresponding author for data requests.

## Abbreviations

ICC: Intraclass correlation coefficient of reliability
CBSM: Community-based social marketing
CVR: Content validity ratio
CVI: Content validity index
DWLS: Diagonal weighted least squares robust estimation
WLSMV: Weighted least squares means and variance adjusted
